# ICU nurses’ knowledge and attitude towards electrocardiogram interpretation in Fujian province, China: a cross-sectional study

**DOI:** 10.3389/fmed.2023.1260312

**Published:** 2023-09-28

**Authors:** Salome E. Buluba, Jinyi He, Hong Li

**Affiliations:** ^1^School of Nursing, Fujian Medical University, Fuzhou, Fujian, China; ^2^Department of Cardiovascular Surgery, Fujian Provincial Hospital, Fuzhou, Fujian, China; ^3^School of Public Health, Fujian Medical University, Fuzhou, Fujian, China

**Keywords:** knowledge, attitude, ICU, nurses, ECG interpretation

## Abstract

**Introduction:**

The series of electrocardiograms (ECGs) can help track cardiac abnormalities in patients’ conditions and make an earlier clinical decision. It is crucial for nurses working in critical care environments to acquire ECG knowledge for effective ECG monitoring and act accordingly in case of a change in patient condition. This study aimed at investigating intensive care unit (ICU) nurses’ knowledge and attitude towards ECG interpretation in Fujian province, China. The study also analyzed the relationship between participants’ demographic characteristics and level of ECG knowledge.

**Methods:**

This study was done online at twenty-one hospitals in Fujian province using a quantitative cross-sectional design involving 357 registered nurses working in the ICU between October and December 2021. The selection of hospitals and potential participants involved purposive and convenient sampling methods, respectively. Binary logistic regression was carried out to determine factors that predict ICU nurses’ knowledge of ECG interpretation, and a *p*-value <0.05 was deemed statistically significant.

**Results:**

The majority of nurses (70.9%) demonstrated a low level of ECG knowledge. The mean score for ECG knowledge was 5.95 (SD = 2.14), with only 0.8% of ICU nurses answering all questions correctly. The majority portrayed positive attitude towards ECG interpretation; however, more than half (61.6%) believed that nurses should rely on a doctor’s opinion about ECG interpretation. Previous ECG training (AOR = 3.98, 95% CI: 2.12–7.45); frequency of ECG interpretation in comparison with no frequency of ECG interpretation (1–3 times per day: AOR = 15.55, 95% CI: 6.33–38.18; 1–3 times per week: AOR = 18.10, 95% CI: 6.38–51.34); and current working unit in comparison to those working in cardiac ICU (general ICU: AOR = 0.45, 95% CI: 0.21–0.94; medical ICU; AOR = 0.28, 95% CI: 0.12–0.67; and surgical ICU; AOR = 0.05, 95% CI: 0.01–0.43) remained statistically significant after adjusting for confounders.

**Conclusion:**

The present study revealed a low level of knowledge about ECG interpretation among ICU nurses. Although the participants demonstrated positive attitudes toward ECG interpretation, the negative attitude still existed. Nurses should acknowledge ECG interpretation as part of their duties and responsibilities in nursing care instead of merely relying on doctors’ opinions.

## Introduction

An electrocardiogram (ECG) is a fast, non-invasive, painless, cheapest, and safe diagnostic tool primarily used at different hospitals in guiding the management of patients with or suspected of cardiac abnormalities (1). An electrocardiogram, commonly abbreviated as EKG or ECG, is defined by the American Heart Association (AHA) as “a test that measures a heartbeat’s electrical activity.” An electrical impulse that is commonly identified as “a wave” travels through the heart with each beat, and as a result, cardiac muscles squeeze and pump blood from the heart (2). The series of ECGs can help track cardiac abnormalities in patients’ conditions and make an earlier clinical decision (1).

The ECG is utilized in patients’ surveillance for cardiac arrhythmias, myocardial ischemia, and QT interval prolongation (3, 4). Therefore, ECG monitoring is highly recommended in patients presenting with chest pain and those with known cardiac risk factors such as patients with stroke, diabetes, acute renal failure, respiratory dysfunction, trauma, drug overdose, sepsis, shock, older adult patients with risk factors of coronary heart disease (5). For closely monitoring, patients with the aforementioned medical conditions are commonly found in critical care units, including the intensive care unit (ICU) or coronary care unit (CCU) (5). Hence, it is crucial for nurses working in these units to acquire knowledge of basic ECG rhythms for effective patient monitoring and act accordingly depending on the information displayed on the cardiac monitor (1, 3, 6, 7). However, cardiac monitors used in most critical care settings display electrical activity of the heart mainly on lead II, thus, a nurse will need to perform a 12-lead ECG to confirm the arrhythmia (6).

Different methods have been put forth to improve nurses’ knowledge about ECG as guidance for easier ECG interpretation when caring for patients needing ECG monitoring. Fred Killingbeck developed an algorithm, the so-called “cardiac rhythm identification for simple people (CRISP) method,” to guide nurses in the interpretation of basic ECG rhythms (8). The latter is the simplest and most effective method for facilitating ECG interpretation accuracy among nurses. The study found a significant increase in accurate interpretation of non-fatal arrhythmias among study participants after the intervention (9). Besides, there had been an online ECG monitoring educational program that aimed at equipping nurses working in cardiac units with knowledge on effective ECG monitoring to improve the quality of nursing care and patient outcomes (3).

Several studies have reported knowledge gaps in ECG interpretation among critical care nurses (10–15). Studies conducted in Sweden among ambulance nurses and Iran among emergency nurses and emergency medical service personnel evidenced that nurses have low ECG knowledge, resulting in poor ECG interpretation competency (12, 16). Ineffective ECG monitoring for cardiac arrhythmias and ischemia due to poor ECG knowledge is linked to increased cases sudden cardiac death (17). However, a study done by Tahboub and Yilmaz revealed a high ECG knowledge level among nurses working in critical care units, namely ICU, emergency department, CCU, cardiology department, and recovery department (15). Previous ECG training, working unit, and nursing working experience have been associated with the level of ECG knowledge in the previous studies (7, 15).

However, very few studies have assessed knowledge about ECG among nurses working in critical care settings (7, 15), where most studies have solely focused on nurses’ practice or competency in ECG interpretation (10–12, 14–16). There is a single study in China mainland about ECG knowledge that was conducted in Wuhan province in the year 2013, which is not recent; nurses’ knowledge about ECG in Fujian province is unknown. Nevertheless, limited studies have evaluated nurses’ attitude towards ECG interpretation. There is a study that reported nurses’ attitude towards continuous ST-segment monitoring (18), but this was very specific. Hence, it is crucial to determine the current level of ECG knowledge and attitude towards ECG interpretation among Chinese nurses working in ICUs; and figure out the need to formulate an ECG education program to improve knowledge level and maximize the quality of nursing care. Therefore, this study aimed at investigating ICU nurses’ knowledge and attitudes toward ECG interpretation in Fujian province, China, as well as analyzing the relationship between participants’ demographic characteristics and level of ECG knowledge.

## Methods

### Study design and setting

Using a quantitative cross-sectional design, this study was carried out online at hospitals in Fujian province, China. A total of twenty-one hospitals from all nine cities that include Fuzhou (5), Quanzhou (2), Xiamen (2), Zhangzhou (2), Putian (2), Longyan (2), Nangping (2), Ningde (2), and Sanming (1) in Fujian province were involved in this study. The hospitals were either secondary or tertiary comprising of comprehensive and specialized ICUs.

### Study population and sampling

This study conveniently recruited registered nurses working in ICUs, namely, general ICU, cardiac ICU, coronary care unit, medical ICU, surgical ICU, and emergency ICU at the selected hospitals between October and December 2021. Being registered with the Fujian Health Commission, having at least 3 months experience of working in a critical care unit, and being willing to take part in the study were the inclusion criteria. While exclusion criteria included being an intern nurse and inactively involved in patient care, like nurse managers and those with more than one month of sick leave. The hospitals were chosen via purposeful sampling since they are adequately suited to care for adult patients with critical illnesses.

### Sample size calculation

The sample size for this study was obtained using a formula for estimating a single population proportion for an infinite population (19). The formula was as follows:


$$n=\frac{z^{2}\;p\;\left(1-p\right)}{d^{2}}$$

Where: n = required sample size.

z = confidence level at 95% (1.96).

p = expected proportion of nurses’ knowledge on ECG, 69% (0.69) (15).

d = confidence interval 5%(0.05).


$$n=\frac{1.96^{2}\;x\;0.69\;\left(1-0.69\right)}{0.05^{2}}=328.69$$

The found value (328.69) needed to be adjusted for non-response, which was set at 10% (20) by the following formula:


$$\frac{\mathrm{Sample}\;\mathrm{size}}{1-\%\mathrm{of}\;non\;\mathrm{response}}=\frac{328.69}{\left(1-10\%\right)}=\frac{328.69}{\left(1-0.1\right)}=365.21$$

Therefore, the calculated sample size was 365, but the study recruited 357 ICU nurses due to their availability.

### Data collection instrument

The researchers developed the tool used to measure variables in this study based on the literature review to meet study objectives (3, 7, 10, 15). Brislin’s back-translation technique for cross-cultural research was used to translate the questionnaire from English into the Chinese language (21). Four bilingual English experts familiar with both English and Chinese languages were consulted. The experts had a mean English teaching/cardiology/ICU working experience of 8.5 years ranging from 6 to 12 years. For cultural adaptation, we consulted six experts specializing in critical care and cardiology to modify the Chinese questionnaire.

The study tool comprised of four sections: section one composed of ICU nurses’ demographic characteristics; ECG interpretation knowledge in section two; ICU nurses’ attitudes towards ECG interpretation in section three; and ECG interpretation practice and confidence in section four. ECG knowledge assessment consisted of 10 true/false questions, and the attitude part consisted of 10 statements in a 5-point Likert scale form. Participants were required to choose either “strongly agree,” “agree,” “Neutral,” “disagree,” or “strongly disagree”.

The Chinese version of the questionnaire was used to ensure the validity and reliability of the tool. Delphi technique was adopted by consulting twenty experts in cardiology and critical care nursing. The experts had a mean (SD) of 20.9 (7.82) years of specialization experience ranging between 6 and 32 years. The positive coefficient was 100%, which is expressed by experts’ response rate of the evaluation; the authority coefficient (Cr) was 0.83, which is determined on the basis of experts’ judgment (Ca) and familiarity (Cs) with the contents; and the coordination coefficient of experts was computed by determining Kendall’s coefficient of concordance (Kendall’s W), 0.11 (*p* < 0.001, *X*^2^ = 71.72) in the first round and 0.1 (*p* = 0.002, *X*^2^ = 57.32) in the second round, thus indicating high consistent opinions in the content evaluation among experts. In checking for tool validity, the computed scale content validity index (S-CVI) average gave a value of 0.97. However, tool reliability was ensured by carrying out a pre-test on 57 ICU nurses, and the computed Cronbach’s Alpha and intra-class reliability coefficient were 0.81and 0.86, respectively.

### Data collection and variables

Wenjuanxing is an online questionnaire system where we uploaded the Chinese version questionnaire.^1^ Participants who met the inclusion criteria were approached and asked to fill in the questionnaire from the generated link that was shared via WeChat, China’s most popular social media platform. We adopted the address restriction technology to ensure that users with the same IP address could only fill in the survey once. Online consent to take part in the study was obtained, and the participants were assured of a high level of confidentiality. Participants were asked to fill out the questionnaire during their free time to avoid interfering with patient care, which did not take more than 15 min.

The variables involved in this study include ICU nurses’ demographic characteristics as independent variables and ICU nurses’ ECG knowledge and attitude towards ECG interpretation as dependent variables. ECG knowledge is referred to as nurses’ comprehension of ECG and its interpretation, that was measured using ten true/false ECG statements; and ECG interpretation attitude is referred to as preconceived beliefs/ideas, feelings, and behavior on accurate ECG monitoring and interpretation, that was measured by thoughts self-rating (5-point Likert scale) on provided ECG interpretation statements.

### Data analysis

Data analysis was done using the Statistical Package for the Social Sciences (SPSS) version 22. Data completeness and consistency were ensured before analysis. Variables for the study were presented as frequencies, percentages, means, and standard deviations. For true/false ECG knowledge questions, a score of 1 was assigned for every correct response and 0 for the incorrect response. For ECG interpretation attitude questions expressed in a Likert scale, the responses were coded from one (1) to five (5), where 1 was regarded as “strongly disagree,” 2 as “disagree,” 3 as “neutral,” 4 as “agree,” and 5 as “strongly agree.” In later analysis, “strongly disagree” and “disagree” were considered to be “disagree,”; and “agree” and “strongly agree” as “agree”; neutral remained as it was. The ECG knowledge part had 10 points as a maximum score, where a mean score of ≥7.5 indicated a high level of ECG knowledge, and that of <7.5 indicated a low level of ECG knowledge (10, 12). Binary logistic regression was carried out to determine factors that predict ICU nurses’ knowledge of ECG interpretation. The degree of variability was tested by Chi-square test, and a *p*-value <0.05 was deemed statistically significant.

### Ethics approval

Ethical approval with Ref. no K2021-08-013 was granted by the Fujian Provincial Hospital Ethics Committee. Permission to conduct the study was obtained from the hospital management of the selected hospitals. In-charges of the ICU departments were also informed about the study. Potential participants were well informed about the study, and online consent was obtained from the willing participants. The latter were assured of confidentiality and anonymity for their participation in the study. Besides, participants were informed about the right to drop off from the study with no negative impact on their career. Code numbers instead of participant names were used for confidentiality purposes during the data collection.

## Results

### Demographic characteristics of the participants

Initially, the study had to involve 365 ICU nurses; however, a total of 357 ICU nurses participated in this study due to their availability, leading to a 97.8% response rate. The majority (81.5%) were females and working in the general ICU (42.3%). Over half (54.1%) were from grade A tertiary hospitals and most nurses (51.5%) were aged 30–39 years. Sixty-two percent of ICU nurses had a bachelor’s degree, with the majority (35.6%) having 6–10 years of working experience in clinical nursing. Nearly half of participants were having 1–5 years of working experience in the critical care setting and 50.4% had no previous ECG training course, and still nearly half (41.2%) had no desire to learn ECG interpretation. For ICU nurses with previous ECG training, the majority (51%) took the course more than 3 years past. The majority (99.7%) reported the internet as the source of ECG training courses, and 36.4% had no frequency of ECG interpretation (Table 1).

**Table 1:** Demographic characteristics of the study participants (*N* = 357).

Variable	Frequency *n* (%)
Age	
20–29 years	143 (40.1)
30–39 years	184 (51.5)
40–50 years	30 (8.4)
Gender	
Male	66 (18.5)
Female	291 (81.5)
Working hospital’s official grade certification	
Grade A level II hospitals	66 (18.5)
Grade B tertiary hospitals	98 (27.5)
Grade A tertiary hospitals	193 (54.1)
Currently working unit	
General ICU	151 (42.3)
Emergency ICU	28 (7.8)
Medical ICU	76 (21.3)
Surgical ICU	30 (8.4)
Cardiac ICU	72 (20.2)
Years of working experience in clinical nursing	
<1 year	2 (0.6)
1–5 years	111 (31.1)
6–10 years	127 (35.6)
11–20 years	97 (27.2)
>20 years	20 (5.6)
Years of working experience in the critical care setting	
<1 year	7 (2.0)
1–5 years	151 (42.3)
6–10 years	110 (30.8)
11–20 years	80 (22.4)
>20 years	9 (2.5)
Education level	
Advanced Diploma	127 (35.6)
Bachelor’s Degree	222 (62.2)
Master’s Degree	8 (2.2)
Job title	
Junior	159 (44.5)
Intermediate certificate	148 (41.5)
Senior	50 (14.0)
Work position	
Head nurse	10 (2.8)
Nursing captain	31 (8.7)
Clinical instructor	29 (8.1)
Clinical nurse	287 (80.4)
Previous ECG training course	
Yes	177 (49.6)
No	180 (50.4)
Years since taking the last ECG course	
<6 months	32 (9.0)
7–12 months	52 (14.6)
13 months-3 years	91 (25.5)
>3 years	182 (51.0)
Source of the ECG training course	
University/School	144 (40.3)
Hospital training	148 (41.5)
Clinical practice	183 (51.3)
Colleague experience	143 (40.1)
Journal/Self-learning books	59 (19.3)
Seminar/Congress/Conferences	94 (26.3)
Internet	356 (99.7)
Others	29 (8.1)
Desire to learn ECG interpretation	
Yes	210 (58.8)
No	147 (41.2)
Frequency of ECG interpretation	
1-3times/day	121 (33.9)
1-3times/week	57 (16.0)
1-3times/month	38 (10.6)
1-3times/year	11 (3.1)
None	130 (36.4)

*n*, number of participants; %, percentage.

### ICU nurses’ ECG knowledge

The mean score (SD) of ECG knowledge was 5.95 (2.14), with only three nurses (0.8%) answering all questions correctly. The majority of ICU nurses (70.9%) demonstrated a low level of ECG knowledge. The statements “QRS complex represented right and left ventricular depolarization” and “If in an ECG the P wave does not appear, there is a conduction problem between the atriums” were the most correctly responded questions, 89.9 and 86.3%, respectively. Besides, the statement that stated, “In Second-degree AV block type II, the PR interval is not constant with conducted beats and some P waves will be present without a QRS complex” was the least correctly responded question (16.2%) (Table 2).

**Table 2:** ECG knowledge among ICU nurses (*N* = 357).

ECG statement	True/False	Correct answers		Mean (SD)
		*n*	%	
The correct order of ECG waves and intervals includes “T wave, P wave, QRS complex, PR interval, ST interval, U wave”.	False	148	41.5	
If in an ECG the P wave does not appear, there is a conduction problem between the atriums	True	308	86.3	
The P wave represented right and left atrial repolarization	False	198	55.5	
QRS complex represented right and left ventricular depolarization	True	321	89.9	
Pathologic Q waves are a sign of previous myocardial infarction	True	245	68.6	
Atrial fibrillation could be a regular rhythm	False	264	73.9	
ECG can detect left ventricular hypertrophy (LVH)	True	217	60.8	
In Second-degree AV block type II, the PR interval is not constant with conducted beats, and some P waves will be present without a QRS complex	False	58	16.2	
Atrial flutter is characterized by a regular ventricular rhythm with a consistent R-R interval	True	189	52.9	
Ventricular fibrillation is characterized by no identifiable P waves and T waves, but the QRS complex is identifiable	False	175	49.0	
Mean score				5.95 (2.14)

*n*, number of participants; %, percentage; SD, standard deviation.

### Attitude towards ECG interpretation

Eighty-six percent of ICU nurses agreed ECG interpretation is difficult. Regarding nurses’ attitudes toward ECG knowledge, the majority (63.9%) agreed not to have adequate knowledge to interpret patients’ ECG readings. Almost half (45.4%) reported not being well trained to interpret patients’ ECG readings during nursing care provision.

Most nurses (68.6%, 70.3%, and 76.8%) demonstrated positive attitudes toward ECG interpretation by disagreeing that “it is minimally important for a nurse working in the ICU to have skills on ECG interpretation”; “ECG interpretation is not meant for nurses”; and “ECG interpretation is not among the priorities in nursing care,” respectively. Besides, nearly all ICU nurses (97.5% and 97.2%) agreed that “knowledge of ECG interpretation among ICU nurses helps with an early diagnosis of a patient with cardiac arrhythmias” and “regular, continuous education about ECG facilitates nurses’ ability to interpret ECG readings,” respectively. However, most ICU nurses (61.6%) portrayed a negative attitude by agreeing with the statement, “I think nurses should rely on a doctor’s opinion about ECG interpretation” (Table 3).

**Table 3:** ICU nurses’ attitude towards ECG interpretation (*N* = 357).

Attitudes	Agree	Neutral	Disagree
	*n* (%)	*n* (%)	*n* (%)
I think ECG interpretation is difficult	327 (86.0)	25 (7.0)	25 (7.0)
I think it is minimally important for a nurse working in the ICU to have skills in ECG interpretation	67 (18.8)	45 (12.6)	245 (68.6)
I think nurses should rely on doctor’s opinions about ECG interpretation	220 (61.6)	58 (16.2)	79 (22.1)
I think knowledge of ECG interpretation among ICU nurses helps with an early diagnosis of a patient with cardiac arrhythmias	348 (97.5)	6 (1.7)	3 (0.8)
I think I have inadequate knowledge to be able to interpret patients’ ECG readings	228 (63.9)	76 (21.3)	53 (14.8)
I think ECG interpretation is not among the priorities in nursing care	34 (9.5)	49 (13.7)	274 (76.8)
I think vital sign monitoring is the most important for critically ill patients in nursing care	353 (98.9)	4 (1.1)	0 (0)
I think ECG interpretation is not meant for nurses	59 (16.5)	47 (13.2)	251 (70.3)
I think regular, continuous education about ECG facilitates nurses’ ability to interpret ECG readings	347 (97.2)	7 (2.0)	3 (0.8)
I think I am not well trained to be able to interpret patients’ ECG readings during nursing care provision	162 (45.4)	107 (30.0)	88 (24.6)

*n*, number of participants; %, percentage.

### Relationship between ICU nurses’ demographic characteristics and the level of ECG knowledge

In bivariate analysis, current working unit (*p* < 0.001), working hospital’s official grade certification (*p* < 0.001), previous ECG training (*p* < 0.001) (hospital training, *p* = 0.036), clinical practice as a source of ECG training course (*p* < 0.001), desire to learn ECG interpretation (*p* < 0.001), and ECG interpretation frequency (*p* < 0.001) had significant associations with the ECG knowledge level (Table 4). Likewise, all the demographics mentioned above showed significant association with the ECG knowledge level in the univariate binary logistic regression analysis (Table 5).

**Table 4:** The association between ICU nurses’ demographics and ECG knowledge.

Variables		High knowledge *n* (%)	Low knowledge *n* (%)	*p*-value
Age				
20–29 years		37 (25.9)	106 (70.1)	0.425
30–39 years		56 (30.4)	128 (69.6)	
40–50 years		11 (36.7)	19 (63.3)	
Gender				
Male		21 (31.8)	45 (68.2)	0.595
Female		83 (28.5)	208 (71.5)	
Working hospital’s official grade certification				
Grade A level II hospitals		34 (51.5)	32 (48.5)	**<0.001**
Grade B tertiary hospitals		21 (21.4)	77 (78.6)	
Grade A tertiary hospitals		49 (25.4)	144 (74.6)	
Currently working unit				
Emergency ICU		8 (28.6)	20 (71.4)	**<0.001**
General ICU		48 (31.8)	103 (68.2)	
Medical ICU		15 (19.7)	61 (80.3)	
Surgical ICU		1 (3.3)	29 (96.7)	
Cardiac ICU		32 (44.4)	40 (55.6)	
Years of working experience in clinical nursing				
<1 year		1 (50.0)	1 (50.0)	0.745
1–5 years		28 (25.2)	83 (74.8)	
6–10 years		37 (29.1)	90 (70.9)	
11–20 years		31 (32.0)	66 (68.0)	
>20 years		7 (35.0)	13 (65.0)	
Years of working experience in the critical care setting				
<1 year		2 (28.6)	5 (71.4)	0.706
1–5 years		43 (28.5)	108 (71.5)	
6–10 years		28 (25.5)	82 (74.5)	
11–20 years		28 (35.0)	52 (65.0)	
>20 years		3 (33.3)	6 (66.7)	
Education level				
Advanced diploma		38 (29.9)	89 (70.1)	0.390
Bachelor’s degree		62 (27.9)	160 (72.1)	
Master’s degree		4 (50.0)	4 (50.0)	
Job title				
Junior		50 (31.4)	109 (68.6)	0.293
Intermediate certificate		44 (29.7)	104 (70.3)	
Senior		10 (20.0)	40 (80.0)	
Work position				
Head nurse		2 (20.0)	8 (80.0)	0.387
Nursing captain		9 (29.0)	22 (71.0)	
Clinical instructor		7 (24.1)	22 (75.9)	
Clinical nurse		86 (30.0)	201 (70.0)		Variables	High knowledge *n* (%)	Low knowledge *n* (%)	*p*-value
Previous ECG training course				
Yes		69 (39.0)	108 (61.0)	**<0.001**
No		35 (19.4)	145 (80.6)	
Years since taking the last ECG course				
<6 months		14 (43.8)	18 (56.3)	0.053
7–12 months		20 (38.5)	32 (61.5)	
13 months-3 years		26 (28.6)	65 (71.4)	
>3 years		44 (24.2)	138 (75.8)	
Source of ECG training course				
University/School	Yes	45 (31.3)	99 (68.8)	0.469
	No	59 (27.7)	154 (72.3)	
Hospital training	Yes	52 (35.1)	96 (64.9)	**0.036**
	No	52 (24.9)	157 (75.1)	
Clinical practice	Yes	37 (20.2)	146 (79.8)	**<0.001**
	No	67 (38.5)	107 (61.5)	
Colleague experience	Yes	40 (28.0)	103 (72.0)	0.693
	No	64 (29.9)	150 (70.1)	
Journal/Self-learning books	Yes	22 (31.9)	47 (68.1)	0.575
	No	82 (28.5)	206 (71.5)	
Seminar/Congress/Conferences	Yes	21 (22.3)	73 (77.7)	0.091
	No	83 (31.6)	180 (68.4)	
Internet	Yes	33 (36.7)	57 (63.3)	0.069
	No	71 (26.6)	196 (73.4)	
Others	Yes	13 (44.8)	16 (55.2)	0.052
	No	91 (27.7)	237 (72.3)	
Desire to learn ECG interpretation				
Yes		77 (36.7)	133 (63.3)	**<0.001**
No		27 (18.4)	120 (81.6)	
Frequency of ECG interpretation				
1-3times/day		59 (48.8)	62 (51.2)	**<0.001**
1-3times/week		32 (56.1)	25 (43.9)	
1-3times/month		4 (10.5)	34 (89.5)	
1-3times/year		2 (18.2)	9 (81.8)	
None		7 (5.4)	123 (94.6)	

*n*, number of participants; %, percentage; bolded values, statistically significant *p* < 0.05.

**Table 5:** Univariate and multivariate logistic regression of factors associated with the level of ECG knowledge among ICU nurses.

Variables		ECG interpretation knowledge			
		COR (95% CI)	*p*-value	AOR (95% CI)	*p*-value
Working hospital’s official grade certification					
Grade A level II hospitals		1		1	
Grade B tertiary hospitals		0.26 (0.13–0.51)	**<0.001**	0.77 (0.29–2.02)	0.588
Grade A tertiary hospitals		0.32 (0.18–0.57)	**<0.001**	0.69 (0.31–1.56)	0.377
Currently working unit					
Emergency ICU		0.50 (0.20–1.28)	0.149	0.43 (0.13–1.37)	0.158
General ICU		0.58 (0.33–1.03)	0.067	0.45 (0.21–0.94)	**0.035**
Medical ICU		0.31 (0.15–0.64)	**0.002**	0.28 (0.12–0.67)	**0.004**
Surgical ICU		0.04 (0.01–0.33)	**0.003**	0.05 (0.01–0.43)	**0.006**
Cardiac ICU		1		1	
Previous ECG training course					
Yes		2.65 (1.64–4.26)	**<0.001**	3.98 (2.12–7.45)	**<0.001**
No		1		1	
Source of ECG training course					
Hospital training	Yes	1.64 (1.03–2.59)	**0.036**	0.73 (0.39–1.38)	0.330
	No	1		1	
Clinical practice	Yes	0.41 (0.25–0.63)	**<0.001**	0.63 (0.33–1.21)	0.164
	No	1		1	
Desire to learn ECG interpretation					
Yes		2.57 (1.56–4.26)	**<0.001**	1.56 (0.81–3.03)	0.184
No		1		1	
Frequency of ECG interpretation					
1-3times/day		16.72 (7.21–38.77)	**<0.001**	15.55 (6.33–38.18)	**<0.001**
1-3times/week		22.49 (8.92–56.66)	**<0.001**	18.10 (6.38–51.34)	**<0.001**
1-3times/month		2.07 (0.57–7.48)	0.268	2.60 (0.68–9.97)	0.164
1-3times/year		3.91 (0.71–21.61)	0.119	2.62 (0.45–15.34)	0.287
None		1		1	

COR, crude odds ratio; AOR, adjusted odds ratio; CI, confidence interval; bolded values, statistically significant *p* < 0.05.

When adjusted with other factors, the ECG interpretation frequency, current working unit, and previous ECG training remained statistically significant with the ECG knowledge level. Nurses working in general, medical, and surgical ICUs had lower odds of having a high level of ECG knowledge compared to nurses working in cardiac ICU (AOR = 0.45, 95% CI: 0.21–0.94; AOR = 0.28, 95% CI: 0.12–0.67; and AOR = 0.05, 95% CI: 0.01–0.43, respectively). The odds of having a high level of ECG knowledge among ICU nurses with previous ECG training were 3.98 times more than those who lacked previous ECG training (95% CI: 2.12–7.45). Also, the odds of having a high level of ECG interpretation knowledge among ICU nurses with 1–3 times a day and 1–3 times a week ECG interpretation frequency were 15.55 and 18.10 times more than those with no ECG interpretation frequency (95% CI: 6.33–38.18; 95% CI: 6.38–51.34, respectively) (Table 5).

## Discussion

The purpose of this study was to investigate ICU nurses’ knowledge and attitudes towards ECG interpretation, as well as the relationship between demographic characteristics and ECG knowledge. ICU nurses had a quite low level of ECG knowledge. Despite the nurses’ positive attitudes toward ECG interpretation, there were still some negative attitudes evident. Besides, predictors for ICU nurses’ level of ECG knowledge included the current working unit, previous ECG training, and ECG interpretation frequency. These study findings manifest a substantial ECG knowledge gap among ICU nurses in Fujian Province.

The overall level of knowledge on ECG interpretation was found to be low in this study. Only 0.8 percent of ICU nurses correctly answered all questions, resulting in a mean knowledge score of 5.95 ± 2.14 (maximum score = 10 points). Only 29.1% of ICU nurses had a high knowledge level, in contrast to an earlier study conducted by Tahboub and Yilmaz, which found that nurses working in critical care settings had a high level of knowledge about ECG interpretation (15). However, the disparity could be explained by differences in knowledge score categorization; these studies did not specify how they classified the level of knowledge as high or low.

Over half of the ICU nurses were unfamiliar with the proper ECG waves and intervals order. The basic ECG knowledge one should gain and crucial in ECG interpretation is to know the correct order of ECG waves and the interval. All nurses (especially those working in the ICU) encountering patients needing ECG monitoring are expected to know the exact order of ECG waves and intervals. Because knowing the exact order of ECG waves and intervals helps in detecting any abnormalities on the ECG reading, allowing an early clinician notification to intervene promptly (5). In contrast, all participants in a previous study by Coll-Badell et al. recognized the correct order for ECG waves and intervals (10). However, the discrepancy might be explained by a variation in the questioning method, with Coll-Badell et al. presenting the question as a multiple-choice question. It probably played a role in recalling the correct order.

Despite having inadequate ECG knowledge, the majority of participants were able to correctly respond to ECG statements such as “The QRS complex represents right and left ventricular depolarization” and “If the P wave does not appear in an ECG, there is a conduction problem between the atriums.” Almost the same percentage of nurses working in critical care environments (86.2 percent) answered the earlier outlined ECG statement correctly in a previous study by Tahboub and Yilmaz (15). However, EMD nurses scored higher (94.7 percent) in the latter ECG statement in a study by Coll-Badell and colleagues (10).

In the current study, the majority of ICU nurses had a negative attitude towards ECG interpretation. Over half of the ICU nurses believed that nurses should rely on doctors’ opinions regarding ECG interpretation. Similarly, Rahimpour et al. found that EMD nurses and EMS personnel believe doctors are the main interpreters of patients’ ECG readings (12). The current study finding denotes that nurses are not well equipped to interpret patients’ ECG readings, which could be aggregated by the lack of ECG knowledge. According to Sangkachand et al., the majority of nurses stated that they did not use ischemia monitoring because of a knowledge deficit (18). Also, the lack of ECG interpretation confidence and the vast diversity in the scope of nursing practice in various hospitals may prompt nurses to assume ECG interpretation is solely the responsibility of doctors (12, 22, 23). In addition, the majority of ICU nurses considered ECG interpretation to be difficult, which could be related to the fact that the majority of ICU nurses lacked ECG training and believed they had insufficient knowledge to interpret patients’ ECG readings. As a result, nurses are unlikely to interpret patients’ ECG strips and assume that ECG interpretation is the role of doctors.

Similar to the study by Zhang and Hsu (7), the present study found the current working unit as a predictor of ECG knowledge. Nurses working in cardiac ICU had higher odds of having a high level of ECG knowledge than nurses from other ICUs (general ICU, medical ICU, and surgical ICU). This finding might be associated with the fact that working in cardiac units exposes nurses to patients who require ECG monitoring on a daily basis. However, ICU nurses from other units should also be familiar with ECG interpretation to prevent further cardiac complications because the majority of patients admitted in the ICU require closely monitoring of different body systems including cardiovascular system. Besides, ICU nurses with daily and weekly ECG interpretation frequency were more likely to have a higher ECG knowledge level than those with no ECG interpretation frequency. This denotes that frequent exposure to ECG interpretation facilitates retention of knowledge. However, ICU nurses with previous ECG training had a higher ECG knowledge level than those with no previous ECG training, similar to a study by Tahboub and Yilmaz (15). The finding explains the significance of continuing education about ECG among nurses working in critical care environments.

The system of nursing education in the Fujian Province is accordance with the national system. The title of registered nurse (RN) is obtained through attaining different level of nursing education including non-traditional postsecondary nursing programs, graduate nursing programs, baccalaureate programs, associate degree programs, and secondary nursing programs (24). Except for graduate nursing programs and non-traditional postsecondary nursing programs, nursing education curriculum includes the professional foundation courses and clinical practice. The ECG teaching is included in physiology or pathophysiology courses and taught in a 1 or 2 class hours during the first year. This may not be maintained in the long term until clinical work, thus call for taking a short course to renew the capability of ECG interpretation. Besides, nurses working in the ICU in China have to undergo a special training on acquiring ICU skills 3 to 6 months under tutor supervision in the clinical area before working independently (25). However, this period of time is not sufficient to acquire all the skills, thus crucial for nurses to be undergoing frequent, continuous education to update their knowledge and improve practice. A national survey had found that most Chinese nurses failed to identify correct electrode placement in cardiac monitoring. This was facilitated by inadequate ECG training among nurses during their undergraduate education and continuous ECG education at work (26). Nonetheless, the current study revealed the significance of previous ECG training course on ICU nurses’ knowledge, where a frequent, continuous ECG education among nurses should be advocated to improve the quality of nursing care.

The present study encompasses some limitations. The study employed convenient sampling in recruiting participants, which limits generalizability. Still, the study involved a larger sample size. The study was at risk of bias because participants could use other sources to get the correct responses. However, they were well informed about the significance of the study in establishing baseline ECG knowledge among nurses working in critical care environments to figure out the need for special demand for ECG training to improve the quality of nursing care and promote better patient outcomes.

## Conclusion

The current study revealed insufficient ECG knowledge among ICU nurses. The strong predictors of ICU nurses’ ECG knowledge level included current working unit, previous ECG training, and frequency of ECG interpretation. Besides, negative attitude towards ECG interpretation was evident from this study, thus, we advise nurses to acknowledge ECG interpretation as part of their duties and responsibilities in nursing care. The collaboration between doctors and nurses in ECG interpretation is vital for better patient outcomes. Nevertheless, this study found a need for frequent continuing education about ECG in Fujian province to improve ICU nurses’ ECG knowledge which will enhance positive attitude towards ECG interpretation, resulting in effective ECG monitoring during nursing care. Also, awareness sensitization on the importance of nurses acquiring ECG knowledge is of great significance among nurses caring for patients needing ECG monitoring because nurses are always first responders to a change in patient condition.

## Data availability statement

The original contributions presented in the study are included in the article/supplementary material, further inquiries can be directed to the corresponding author.

## Ethics statement

The studies involving humans were approved by the Fujian Provincial Hospital Ethics Committee, Fujian Provincial Hospital, Fujian, China. The studies were conducted in accordance with the local legislation and institutional requirements. Written informed consent for participation was not required from the participants or the participants’ legal guardians/next of kin because The study was conducted online, thus, online consent was obtained.

## Author contributions

SB: Conceptualization, Formal analysis, Investigation, Methodology, Validation, Writing – original draft, Writing – review & editing. JH: Investigation, Supervision, Validation, Writing – review & editing. HL: Conceptualization, Supervision, Validation, Writing – review & editing.

## Funding

The author(s) declare financial support was received for the research, authorship, and/or publication of this article. The publication of this article was funded by Fujian Medical University.

## Conflict of interest

The authors declare that the research was conducted in the absence of any commercial or financial relationships that could be construed as a potential conflict of interest.

## Publisher’s note

All claims expressed in this article are solely those of the authors and do not necessarily represent those of their affiliated organizations, or those of the publisher, the editors and the reviewers. Any product that may be evaluated in this article, or claim that may be made by its manufacturer, is not guaranteed or endorsed by the publisher.

## Footnotes

^1^ www.wjx.cn
